# 

**Published:** 1887-06

**Authors:** 


Page.

Original Contributions:
Hydrophobia Vera, with report of a case. J. I. Tucker ... 545

Editorial:
Photography and Medicine ... 550
Chest Movements of Respiration in the Indian Female ... 551

Foreign Correspondence:
Notes from a London Clinic ... 553

Medical Progress:
Hearing and Examinations for the Public Service ... 556
Diet and Hygiene in the Treatment of Diabetes ... 558
Thymol in the Treatment of Atrophic Nasal Catarrh ... 562
The Poisonous Action of Potassium Chlorate ... 569
In How Many Ways Do We Hear ... 571
The Gonococcus of Neiser ... 573
The Differential Diagnosis of Disease of the Sound-Conducting and of the Sound-Perceiving Apparatus ... 575

Society Reports:
*Illinois State Medical Society; Annual Meeting:*
Session of Tuesday, May 17th ... 577
Session of Wednesday, May 18th ... 595
Session of Thursday, May 19th ... 596
*Chicago Medical Society; Stated Meeting of April 18th:*
Burgeon's Method of Treating Phthisis by Gaseous Enemata ... 604
*Chicago Society of Ophthalmology and Otology; Meeting of February 8th:*
The Adjustment of Glasses in Errors of Refraction ... 618
The Galvanic Current of Electricity in in the Treatment of Certain Forms of Cataract ... 620
Case of Partial Trichiasis Relieved by Stellwag's Method ... 620

Clinical Reports:
Aphasia ... 622
Cancer in the Testicle and Effusion in the Left Pleural Cavity ... 625

Extracts:
Honors to a Worthy Practitioner ... 627
Electricity in the Treatment of Epilepsy 627

Book Reviews:
Outlines of the Pathology aud Treatment of Syphilis and Allied Diseases ... 629
Drug Eruptions: A Clinical Study of the Effect of Drugs upon the Skin ... 630
Elementary Microscopical Technology: A Manual for Students ... 631
Medical Electricity: Its Application to Medicine and Surgery ... 631
Transactions of the Association of American Physicians ... 631
A Treatise on Obstetrics ... 632
Fevers: Their History, Etiology, Diagnosis, Prognosis and Treatment ... 632
A Practical Treatment on Obstetrics ... 633
A Treatise on Diseases of the Skin, with Special Reference to their Diagnosis and Treatment ... 633
A Text Book of Pathological Anatomy and Pathogenesis ... 633

Pamphlets Received ... 635

Index to Volume LIV ... 637

